# The Gentle Side of the UPS: Ubiquitin-Proteasome System and the Regulation of the Myogenic Program

**DOI:** 10.3389/fcell.2021.821839

**Published:** 2022-01-20

**Authors:** Hugo C. Olguín

**Affiliations:** Laboratory of Tissue Repair and Adult Stem Cells, Department of Cellular and Molecular Biology, Faculty of Biological Sciences, Pontificia Universidad Católica de Chile, Santiago, Chile

**Keywords:** ubiquitin (Ub), proteasome, satellite cells, adult stem (AS) cells, stem cell fate, skeletal muscle, myogenesis, myogenic program

## Abstract

In recent years, the ubiquitin-proteasome system (UPS) has emerged as an important regulator of stem cell function. Here we review recent findings indicating that UPS also plays critical roles in the biology of satellite cells, the muscle stem cell responsible for its maintenance and regeneration. While we focus our attention on the control of key transcriptional regulators of satellite cell function, we briefly discuss early studies suggesting the UPS participates more broadly in the regulation of satellite cell stemness and regenerative capacity.

## Introduction

Comprising ∼40% of the total body mass in humans, skeletal muscle (SKM) plays critical roles in locomotion, body posture and whole-body metabolism. Represented by > 600 individual muscles, at the basic level SKM is constituted by bundles of elongated syncytial cells named myofibers. Each myofiber is surrounded by a basal lamina and its sarcoplasm is filled with highly organized myofibrils, formed primarily by end-to-end repetitions of contractile molecular units or sarcomeres (Mukund and Subramaniam, 2019). SKM is a highly plastic tissue endowed with a remarkable regenerative capacity, which is sustained by tissue-specific stem cells known as satellite cells (SCs). These muscle progenitors lie quiescent between the myofiber’s plasma membrane and the basal lamina (Mauro, 1961; Schultz et al., 1978). Upon local or systemic stimuli, SCs become activated, entering the cell cycle giving rise to lineage-committed progenitors that will differentiate and fuse to form new myofibers (Schultz and Jaryszak, 1985; Dumont et al., 2015). As bonafide adult stem cells, SCs are capable of self-renewal, ensuring tissue maintenance and regeneration over time (Zammit et al., 2004; Collins et al., 2005). In this context, a balance between stemness and differentiation is crucial for SC function and several extrinsic and intrinsic pathways have been described to regulate these processes. In recent years, the role of protein homeostasis (proteostasis) in the regulation of stem cell function has become an exciting area of research as many studies suggest this process is particularly robust in embryonic, pluripotent and adult stem cells, while loss of proteostasis affects stemness and differentiation potential in pathological conditions and during aging (Vilchez et al., 2014; Lee et al., 2017; Fernando et al., 2018; Llamas et al., 2020). Among different mechanisms regulating protein homeostasis, the ubiquitin-proteasome system (UPS) is one of the best described. In the context of skeletal muscle tissue, UPS is usually related to loss of muscle mass trough degradation of sarcomeric proteins during atrophy (Combaret et al., 2009; Bodine and Baehr, 2014; Bilodeau et al., 2016; Kitajima et al., 2020), while its role in the regulation of SCs function has received little attention. Here we will briefly describe general aspects of SCs and UPS biology to then focus on recent findings describing UPS-dependent regulation of SC stemness and differentiation. Finally, we will discuss potential new directions for this emerging field in SC research.

### Transcriptional Regulation of Satellite Cell Fate

The myogenic program is orchestrated by a family of transcription factors known as Muscle Regulatory Factors (MRFs; MyoD, Myf5, Myogenin and MRF4). Although MRF proteins contain highly conserved DNA binding motifs, they also exhibit divergent regulatory regions and partially overlapping expression patterns that can explain their hierarchical contribution to muscle formation: while MyoD and Myf5 are required for lineage commitment, Myogenin and MRF4 drive terminal muscle differentiation (Rawls et al., 1998; Zammit, 2017). Since the cellular unit in skeletal muscle is a multinucleated cell arising from myoblast fusion, the temporal regulation of cell division and differentiation is critical for successful muscle formation. In this context, MyoD has been shown to directly activate genes involved in cell cycle progression, thus leading to myoblast proliferation (Zhang et al., 2010; Singh and Dilworth, 2013). In contrast, Myogenin activates genes that switch off cell proliferation, leading myoblasts to exit the cell cycle, engaging in terminal differentiation (Liu et al., 2012). Thus, while MyoD and Myogenin synergize to drive myogenesis, it is the induction of Myogenin that imposes the non-reversible nature of terminal muscle differentiation. During development, muscle growth is determined by myoblast fusion, while postnatal muscle growth is achieved mainly by hypertrophy (i.e., increase of individual myofiber volume). At perinatal stages, myogenic progenitors expressing the transcription factor Pax7, gradually exit the cell cycle into quiescence, forming the original pool of SCs. Upon activation, SCs express and accumulate Myf5 and MyoD proteins. Following a transient phase of robust cell division, these lineage-committed progenitors (adult myoblasts) eventually express the Myogenin protein, leading to new myofiber formation and/or myofiber repair (Ciciliot and Schiaffino, 2010; Wang and Rudnicki, 2011; Dumont et al., 2015).

Critical for the maintenance of their stem cell function, SCs must balance self-renewal with the production of differentiated progeny (Giordani et al., 2018). The molecular regulation of SC maintenance and renewal, however, has recently begun to unveil. In this regard, the transcription factor Pax7 is required for SC specification and function (Seale et al., 2000; Kassar-Duchossoy et al., 2005; Relaix et al., 2005; Günther et al., 2013; von Maltzahn et al., 2013). Pax7 is also involved in cell-identity specification in the pituitary (Budry et al., 2012). Interestingly, both in the pituitary and in muscle stem cells, Pax7 binding to DNA correlates with chromatin modifications associated to changes in gene expression required for lineage commitment and differentiation, acting as a “pioneer transcription factor” (Carrió et al., 2016; Lilja et al., 2017; Mayran et al., 2018). Evidence from our group and others indicate that Pax7 play dual roles in SCs: While it can initiate the myogenic program by inducing *MyoD* and/or *Myf-5* transcription (Gros et al., 2005; Chen et al., 2006; Relaix et al., 2006; McKinnell et al., 2008), it can also repress MyoD activity and the induction of terminal differentiation (Olguin and Olwin, 2004; Olguin et al., 2007; Kumar et al., 2009). In this context, controlling Pax7-to-MyoD protein ratio appears to regulate SC fate. MyoD and Myogenin protein levels are known to be tightly regulated by the ubiquitin-proteasome system (UPS) (Tintignac et al., 2000; Floyd et al., 2001; Lingbeck et al., 2005; Tintignac et al., 2005; Shiraishi et al., 2007; Noy et al., 2012). Interestingly, Pax7-dependent repression of MyoD activity involves MyoD degradation via the UPS.

### Ubiquitin-Proteasome System

**FIGURE 1:**
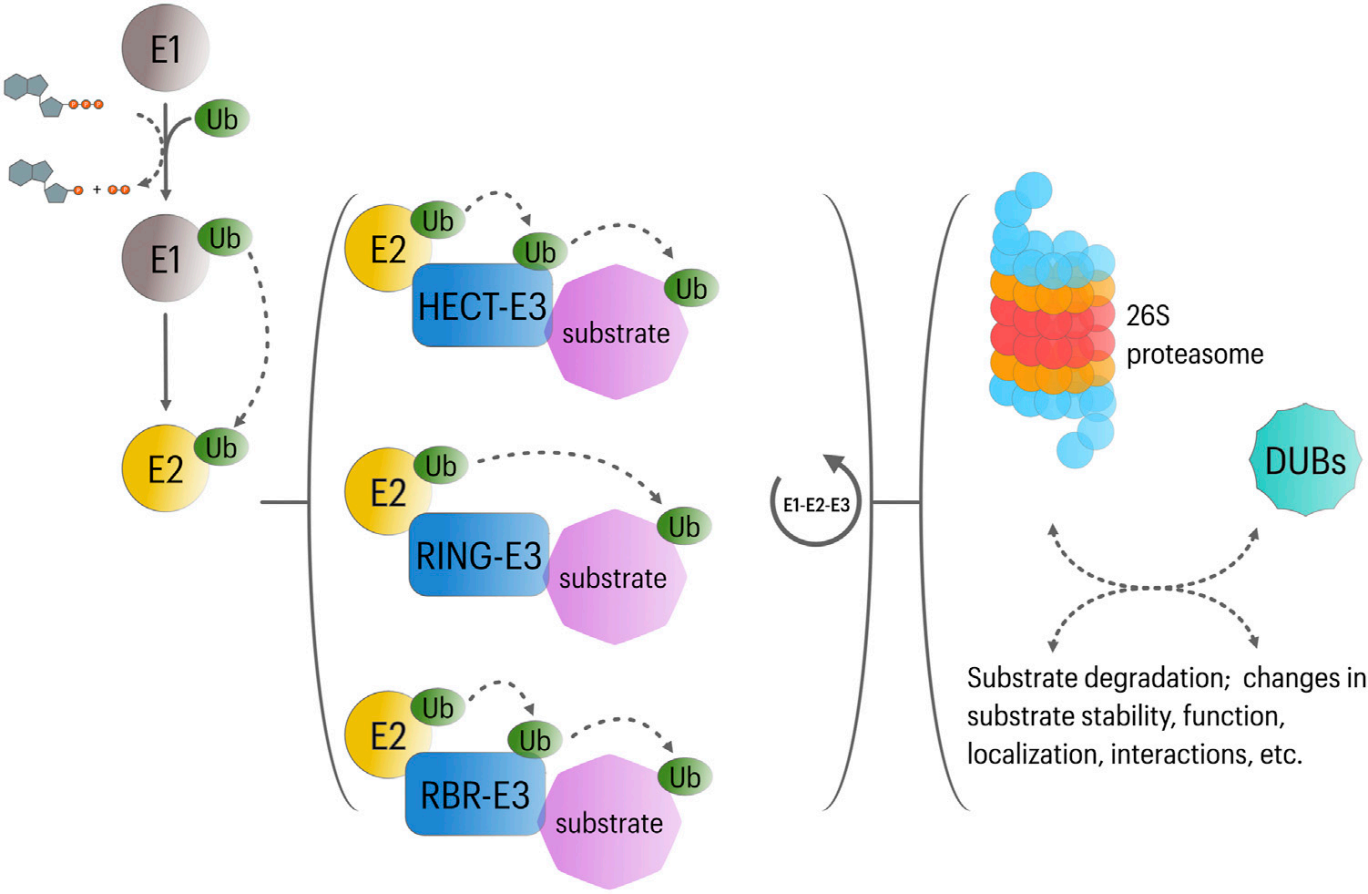
Overview of UPS function. In an ATP-dependent reaction, Ubiquitin is first attached to a cysteine residue of ubiquitin activating enzyme (E1) in an ATP-dependent reaction. Subsequently, the activated ubiquitin transferred to a cysteine residue of conjugating enzyme (E2). Then, along with a specific ubiquitin ligase (E3), E2 participates in the transfer of ubiquitin to a lysine residue of the substrate protein. Three major classes of E3 ligases have been described, including RING-E3, HECT-E3, and RBR-E3 ligases. While RING-E3 catalyze the transfer of ubiquitin directly from E2 ligases to the substrate; activated ubiquitin is first transferred to HECT or RBR-E3 ligases, which then directly ubiquitinate their substrates. Repetition of this cycle is involved in the formation of polyubiquitin chains; however, substrates can be subject to mono-uniquitination or multi-mono-ubiquitination. These ubiquitination modes affect substrate function in different manners. Likewise, different types of polyubiquitin chains appear to regulate different cellular functions: Lys48-linked polyubiquitin chains usually target proteins for proteasomal degradation in different cellular contexts, whereas Lys63, Lys27, and Lys11-linked polyubiquitin chains are implicated in the regulation of NFκB signaling, mitophagy and cell cycle progression, respectively. Finally, deubiquitinating enzymes (DUBs) can determine the fate of ubiquitinated substrates by removing or editing (poly) ubiquitin modifications. DUBs are also involved in ubiquitin recycling.

The UPS mediates intracellular protein degradation in eukaryotic cells with extreme substrate specificity and is therefore crucial for a variety of cellular processes including control of protein quality, transcription, DNA repair, cell cycle progression, stress response and programed cell death (Glickman and Ciechanover, 2002). Protein ubiquitination involves the covalent attachment of the highly conserved protein ubiquitin to lysine residues of substrate proteins, which then acts as a molecular mark for downstream regulatory interactions (Pickart and Fushman, 2004; Trempe, 2011). Ubiquitination requires the activity of three major set of proteins: 1) E1 ubiquitin-activating enzyme, 2) E2 ubiquitin-conjugating enzyme, and 3) E3 ubiquitin ligase (Pickart, 2001). Activated ubiquitin is transferred to the target protein by the ubiquitin E3 ligase, which can additionally catalyze the formation of polyubiquitin chains. While E1 and E2 enzymes are highly conserved, each member of the large superfamily of E3 ligases targets a defined set of proteins. Ubiquitinated proteins are generally destined for degradation by the 26S proteasome; however, ubiquitination can also regulate protein function through non-degradative mechanisms (Woelk et al., 2007) (Figure 1). The diversity of regulatory outcomes depends partially on the type of ubiquitination modifications (i.e., polyubiquitination, monoubiquitination or multi-monoubiquitination) and the ubiquitin lysine residue (K6, K11, K27, K29, K33, K48 or K63) involved in polyubiquitin chain formation. In this context, K48-linked ubiquitin chains were described initially as signal for proteasome degradation, while K11-linked ubiquitin chains and monoubiquitination appeared to be mostly involved in non-degradative functions (Akutsu et al., 2016).

The 26S proteasome is an ATP-dependent protease complex, composed by the 20S proteasome (core complex) and the 19S proteasome cap (regulatory complex). The 20S proteasome exhibits a barrel-shaped structure, formed by the arrangement of two end *α* subunits rings, and two *β* subunits middle rings, bearing proteolytic activity. At the 19S cap, proteins containing ubiquitin-binding domains, such as Rpn10 and Rpn13, function as receptors for ubiquitinated substrates. The 19S cap also contains a central ring structure formed by six AAA ATPases (Rpt1–Rpt6), responsible for substrate binding, unfolding and transfer through the proteasome channel. The *α* subunits bind and guide unfolded target proteins prior to their cleavage into 3–25 aa peptides. These are later cleaved to single amino acids by peptidases, allowing amino acid reutilization by the cells (Tanaka, 2009; Tomko and Hochstrasser, 2013; Bard et al., 2018).

Protein ubiquitination is a dynamic process that can also be modified by ubiquitin proteases commonly referred to as deubiquitinating enzymes (DUBs), which are involved in ubiquitin maturation (Grou et al., 2015), removal (Finley, 2009; Lee et al., 2011), editing of polyubiquitin chains (Newton et al., 2008), and the control of the available pool of free ubiquitin in cells. At least 90 DUBS have been identified in the human genome and grouped into five families according to the conservation of their catalytic domains (Reyes-Turcu et al., 2009).

## UPS and the Regulation of Satellite Cell Function

### Proteasome

Several studies indicate that increased proteostasis is a stem cell hallmark, which is gradually lost upon differentiation (Llamas et al., 2020). Specifically, increased proteasome activity in human embryonic stem cells (hESCs) is required to maintain both pluripotency and their ability to differentiate into specific cell lineages (Vilchez et al., 2014). A follow-up study described that increased proteasome activity in hESCs correlated with increased expression of specific ubiquitin ligases, such as HERC2, UBE3A, UBR7 and RNF181. Characterization of their activities and protein-protein interactions further supported a link with stem cell identity. Interestingly, the authors also suggest that inhibition of specific ubiquitin ligases impaired different cellular processes including transcriptional regulation, protein synthesis, glycolytic metabolism, telomere maintenance and rRNA maturation (Saez et al., 2018).

In an elegant study, Kitajima and cols (Kitajima et al., 2018) recently showed that SC-specific proteasome dysfunction induced by ablation of the Rpt3 26S proteasome subunit, impaired SC function *in vitro* and *in vivo*. Rpt3 appears to be required for degradation of most proteasomal substrates and *Rpt3*-deficient mice embryos die before implantation due to defective blastocyst development (Sakao et al., 2000). Upon induced muscle injury, Rpt3-null SCs exhibited a dramatic decrease in proliferation, that was accompanied by a significant increase in apoptosis correlating with the up-regulation of p53 expression. As expected, these effects resulted in a severely impaired muscle regeneration and reduction of the SC pool. Interestingly, Rpt3-null primary myoblasts showed a marked decrease in differentiation potential, suggesting that proteasome function is critical at different stages of the myogenic progression (Kitajima et al., 2018). Moreover, muscle specific Rpt3 deletion results in severe muscle growth defects, decreased forced generation and the accumulation of abnormal proteins (Kitajima et al., 2014), further supporting a more complex role of the UPS during the formation and maintenance of muscle tissue.

### Ubiquitin Ligases

Including >600 predicted members in humans, E3 ubiquitin ligases (E3s) represent a critical component in the ubiquitination cascade, controlling its efficiency and specificity. According to their structure and the mechanisms involved in ubiquitin transfer, three groups can be recognized within the E3 family. First, a group containing the *homologous to the E6AP carboxyl terminus* (HECT) domain. This domain allows the E3 to accept ubiquitin from an E2 enzyme and to transfer it to a substrate. While the HECT domain is localized to the C-terminus, the N-terminus is involved in substrate recognition (Wang et al., 2020). The second and largest group (∼90% of all described E3s) contains the *Really Interesting New Gene-finger* (RING) domain. RING E3s serve as a platform for both the E2 and the substrate and promote the ubiquitin transfer from the E2 to the target protein. In some multi-protein RING-E3 complexes, different proteins containing protein-protein interaction motifs are involved in substrate recognition (Bulatov and Ciulli, 2015). The third group is known as the *RING-in-Between-RING* (RBR) E3 ligases, and exhibit features of RING and HECT E3s. They utilize an E2-binding RING domain and a second domain (RING2) that binds ubiquitin, which is then transferred to the target protein (Dove and Klevit, 2017) (Figure 1).

Stemness and differentiation are, ultimately, under the precise control of gene expression programs (Takahashi and Yamanaka, 2016; Almeida et al., 2021; Islam et al., 2021). The initiation and maintenance of specific gene expression patterns is achieved by specific combinations of transcriptional regulators, subjected to a variety of post translational modifications controlling their activity, molecular interactions, sub cellular localization and half-life. In this context, several mechanisms involving the UPS have been found to control myogenesis by targeting MyoD function. Specifically, the SCF^MAFbx^ complex (SCF, Skp1, Cdc53/Cullin 1, F-box protein) was first shown to mediate MyoD ubiquitination at lysine 133, followed by proteosomal-dependent degradation (Tintignac et al., 2005). The specificity of SCF towards MyoD depends on the F-box protein atrogin-1/MAFbx, which is also involved in muscle-specific protein degradation during atrophy. In the myogenic cell line C2C12, atrogin expression was shown to increase during differentiation, correlating with down-regulation of MyoD protein levels.

Multiple post translational modifications can promote or prevent ubiquitination, allowing precise fine tuning of transcription factor half-life. In this regard, MyoD can be phosphorylated at S200 by CDK activity, promoting its degradation by the UPS (Kitzmann et al., 1998; Song et al., 1998). MyoD is also subjected to methylation by the G9a methyltransferase, which promotes MyoD ubiquitination by the Cul4/Ddb1/Dcaf1 complex, followed by proteasome-dependent degradation. Conversely, Jmjd2C demethylase prevents MyoD degradation, increasing its transcriptional activity (Jung et al., 2015).

HUWE1 (HECT, UBA and WWE domain containing 1) was also shown to induce MyoD degradation *via* direct ubiquitination *in vitro* and upon over-expression in C2C12 cells (Noy et al., 2012). Interestingly, HUWE1 appears to target several lysine residues in MyoD protein, depending on the phosphorylation status of MyoD. The relevance of this regulation for SC fate remains to be determine, since HUWE1 expression has been shown in post mitotic muscle cells (Liu et al., 2019), and its expression in quiescent or proliferating SCs is yet to be characterized.

Upon activation, proliferating muscle progenitors co-express Pax7 and MyoD at different levels (Casar et al., 2004). As discussed previously, Pax7-to-MyoD ratio could determine SC fate decisions: while SC-specific ablation of Pax7 results in precocious differentiation, Pax7 over-expression inhibits differentiation by inducing MyoD proteasome-dependent degradation (Olguin and Olwin, 2004; Olguin et al., 2007; Lepper et al., 2009). As shown by Joung and cols. (Joung et al., 2014), the Ret finger protein (RFP/TRIM27) E3 ligase can interact with both Pax7 and MyoD (which is ubiquitinated by RFP), mediating the Pax7-induced down-regulation of MyoD. RFP expression in muscle fibers is increased during denervation-induced atrophy, while RFP-null mice exhibited significantly less reduction in muscle mass upon denervation. Noteworthy, RFP-null muscles showed increase numbers of MyoD positive nuclei following denervation (Joung et al., 2014).

Pax7 expression is maintained in muscle progenitors that exit the cell cycle evading the differentiation program. *In vitro*, this population is known as “reserve cells”, since they can re-enter the cell cycle under proper conditions, giving rise to new differentiation-competent cells as well as reserve cells (Yoshida et al., 1998). Therefore, it has been postulated that they may represent an inherent self-renewal mechanism associated to the differentiating population of muscle progenitors (Olguin and Olwin, 2004). In this context, Bustos and cols (Bustos et al., 2015) showed that the HECT E3 ligase Nedd4-1 (neural precursor cell expressed, developmentally down-regulated 4) ubiquitinates Pax7 followed by proteasome-dependent degradation in differentiating C2C12 cells. Nedd4-1 can shuttle between the cytoplasm and the nucleus where it binds and ubiquitinates Pax7. However, in myofiber-associated SCs, Nedd4-1 nuclear import was prevented during the first 24 h after isolation and was only observed after 48 h in culture. These observations suggest that regulation of Nedd4-1 activity and/or sub-cellular localization could be part of the mechanism involved in the control of Pax7 protein levels during the myogenic progression.

Pax7 is also subjected to different post translational modifications. Pax7 is phosphorylated by CK2 (casein kinase 2) in proliferating muscle progenitors and inhibition of CK2 or mutation of the target motif in Pax7, affects Pax7 stability (González et al., 2016). It remains to be determined if CK2 phosphorylation antagonizes Nedd4-1 ubiquitination, however these findings suggest that different mechanism fine tune Pax7 stability to allow muscle differentiation or SC self-renewal. Noteworthy, CK2 phosphorylation motif overlaps with caspases recognition sites (Duncan et al., 2011; Dix et al., 2012) and CK2-dependent phosphorylation prevents Pax7 cleavage by caspase-3 and has been proposed as another mechanisms controlling SC fate via Pax7 degradation (Olguin 2011; Dick et al., 2015).

Pax3, a paralog of Pax7 is also expressed in a subset of SCs (Relaix et al., 2006) which recent studies suggest are resistant to diverse environmental stressors (Der Vartanian et al., 2019; Scaramozza et al., 2019). Pax3 expression in SCs appears to be transient and its protein levels are regulated by monoubiquitination of lysine 475 followed by proteasomal-dependent degradation (Boutet et al., 2007). Taf1, a major subunit of the TFIID transcriptional initiation complex exhibiting both E1 and E2 activities, appears to be necessary and sufficient to promote Pax3 ubiquitination (Boutet et al., 2010).

Induction of Myogenin expression is a hallmark in the path to terminal muscle differentiation. As shown for MyoD, Myogenin protein levels are also regulated by the UPS. Fu and cols. (Fu et al., 2007). demonstrated that a Cullin ubiquitin ligase complex containing the von Hippel-Lindau (VHL) protein, targeted Myogenin for proteasomal degradation. The authors also showed that EGLN3 protein (member of the EGLN family of prolyl hydroxylases) is up regulated during C2C12 differentiation and prevented Myogenin ubiquitination and degradation by inhibiting its interaction with VHL, which acts as a substrate adaptor in the Cullin complex. Interestingly, Huang and cols (Huang et al., 2016) showed that 4.1R protein (component of the contractile apparatus in skeletal muscle) is expressed in differentiating C2C12 cells, where enhances Myogenin stability by binding to VHL, in a similar fashion to EGLN3. Myogenin is also ubiquitinated by the SCF (Skp1-Cullin1-F-box protein)-complex stimulating its degradation in C2C12 cells, while the TBP-interacting protein 120 (TIP120/CAND) can bind to cullin one inhibiting SCF-complex/myogenin interaction; this prevents myogenin ubiquitination, enhancing its stability and C2C12 differentiation (Shiraishi et al., 2007).

In addition to the control of the myogenic transcription factors, the UPS can regulate SC function at different levels. As an example, mutations in the gene coding for the RING- E3 ligase Tripartite motif-containing protein 32 (TRIM32) are associated with limb-girdle muscular dystrophy 2H (LGMD2H) and sarcotubular myopathy (STM) (Frosk et al., 2002; Johnson et al., 2019). Recently, Servián-Morilla and cols (Servián-Morilla et al., 2019) studied primary myoblasts obtained from patients diagnosed with a form of muscular dystrophy and carried different mutations in the TRIM32 gene (resulting in reduced levels of TRIM32 protein). Primary myoblasts exhibited impaired proliferation and differentiation, which was attributed to signs of premature senescence. These findings partially correlate with previous studies in mice, where absence of TRIM32 was associated with accumulation of c-Myc and subsequent inhibition of differentiation (Nicklas et al., 2012). Interestingly, human myoblasts affected by TRIM32 mutations also exhibited increased levels of autophagic flux markers and accumulation of autolysosomes, suggesting that accumulation of potential TRIM32 targets is also involved in differentiation impairment.

At the crossroad between pro-myogenic signaling and chromatin remodeling, the E3 ligase Praja1 (PJA1) ubiquitinates the enhancer of zeste homolog 2 (EZH2) subunit of the Polycomb repressive complex 2 (PRC2), which functions to maintain muscle-specific genes in a repressed state. In this context, p38-alpha kinase (induced upon myogenic stimuli) phosphorylates EZH2 at threonine 372, which is then ubiquitinated by PJA1 followed by proteasome-dependent degradation (Consalvi et al., 2017). The authors also proposed that p38/PJA1-induced degradation of EZH2 triggers a positive feedback loop to down-regulate EZH2 expression.

### Deubiquitinases

∼100 DUBs have been identified in the human genome (Reyes-Turcu et al., 2009) and are grouped in five families according to the conservation of their catalytic domains: ubiquitin C-terminal hydrolase (UCH), ubiquitin specific protease (USP/UBP), ovarian tumor domain (OTU), Josephin domain (MJD) and JAB1/MPN/Mov34 metalloenzyme (JAMM) domain zinc-dependent metalloprotease family.

The role of DUBs activity in the regulation of stem cell fate has been described in different cellular contexts, particularly during early development and gametogenesis (Chandrasekaran et al., 2017); however, the participation of DUBs in the regulation of SC function remains largely unexplored. Nonetheless, Agrawal and cols. (Agrawal et al., 2012). showed that the ubiquitin-specific protease 9, X-linked (USP9X), regulates the Mammalian Target of Rapamycin (mTOR) during myoblast differentiation. Given its role in integrating growth factor and nutrient status with cell growth and metabolism, various studies show mTOR as a critical regulator of muscle cell differentiation and regeneration (Rodgers et al., 2014; Zhang et al., 2015a; Rion et al., 2019; Lund-Ricard et al., 2020). USP9X appears to interact with mTOR in C2C12 cells and functions as a negative regulator of mTORC1 activity and muscle differentiation. Despite the implications of these findings for the regulation of muscle growth and aging, it remains to be determined if enzymatic activity is required for USP9X effect and which mechanim(s) regulate USP9X function. Similarly, Yun and Kim. (2017) reported that USP4 suppressed differentiation in C2C12 myoblasts. Since a catalytically inactive mutant form of USP4 had similar effects, the authors concluded that anti-myogenic activity is independent of DUB activity and correlates with impaired MyoD function and suppression of p38 activity; nonetheless further studies are needed to define the molecular mechanism underlaying these phenomena.

A pro-myogenic effect of DUB activity was recently reported by De la Vega and cols. (de la Vega et al., 2020). The authors showed that Myogenin was deubiquitinated by USP7, favoring Myogenin accumulation in differentiating cells. Accordingly, acute pharmacological inhibition of USP7 activity resulted in impaired muscle regeneration in mice. Interestingly, USP7 expression appears to be transient in adult primary myoblast and during muscle regeneration, correlating with the timing when increase in Myogenin levels and cell fusion are observed. These results suggest that pathways regulating USP7 expression could be important determinants for myogenic progression.

## Discussion and Future Directions

**FIGURE 2:**
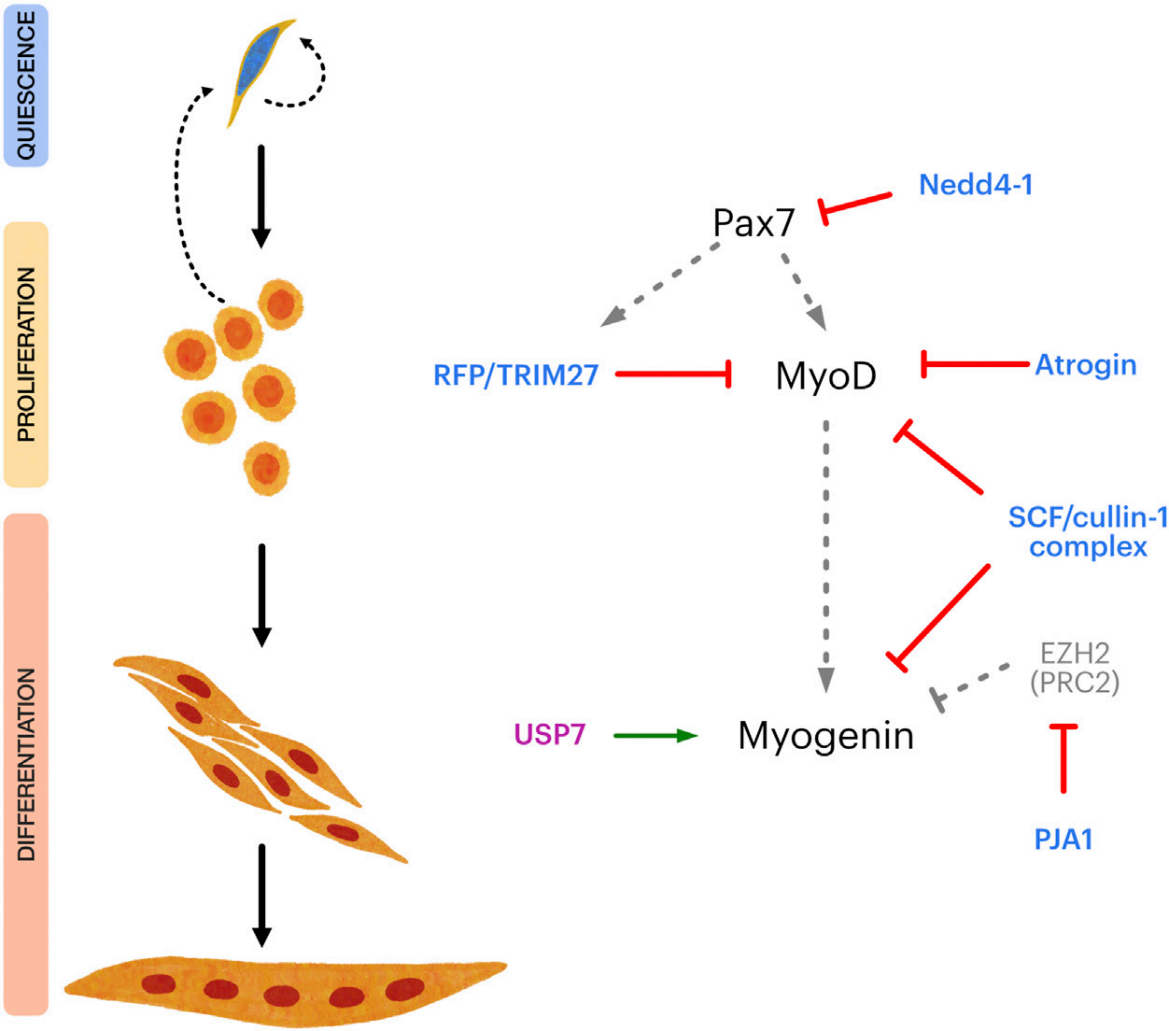
Overview of E3 and DUB regulation of the transcriptional control of satellite cell myogenic progression. Left panel shows different steps in satellite cell myogenic progression, from quiescence to terminal differentiation. Right panel shows key transcriptional regulators of satellite cell maintenance, proliferation, myogenic commitment, and differentiation (Pax7, MyoD and Myogenin, respectively) and different E3 ligases (light blue) and DUBs (magenta) which have been shown to directly regulate them. Grey dotted arrows indicate transcriptional regulation. Red lines indicate negative regulation. Green arrow indicates positive regulation. EZH2 is a subunit of the Polycomb repressor complex 2 (PRC2), negatively regulating Myogenin expression.

The UPS-related mechanisms discussed above control SC fate by targeting key transcriptional regulators (Figure 2). Nonetheless, a variety of relevant E3/DUBs substrates are expressed in SCs that control specific aspects of their myogenic progression, self-renewal, and maintenance of the quiescent state. For example, the E3 ligase Nedd4-1 function as negative regulator of Notch-1 signaling (Koncarevic et al., 2007), regulates FGF receptor endocytosis (Persaud et al., 2011) and favors IGF receptor and insulin receptor signaling by ubiquitinating IGFR-substrate 2 (IRS-2) and phospho-AKT (Cao et al., 2008; Fan et al., 2013; Fukushima et al., 2015; Zhang et al., 2015b). Recent studies indicate that Nedd4-1 is also an important regulator of autophagy (Lin et al., 2017; Sun et al., 2017; Xie et al., 2020a; Xie et al., 2020b). In the context of SC function, autophagy is critical to preserve their myogenic potential during aging as well as for the maintenance of quiescence (García-Prat et al., 2016; Sousa-Victor and Muñoz-Cánoves, 2016; García-Prat and Muñoz-Cánoves, 2017). Although these findings point to the role of autophagy in general terms, is worth noting that autophagy-dependent mitochondrial remodeling (mitophagy) is critical for the myogenic progression, and subject to Nedd4-1 regulation (Lin et al., 2017; Sun et al., 2017). Since Nedd4-1 is expressed in quiescent SCs and throughout their myogenic progression, it is likely to have multiple relevant targets controlling SC fate which remain to be defined.

On the other hand, USP7 is a key regulator of p53 stability, mainly through deubiquitination of murine double minute 2 (Mdm2), the E3 ligase which targets p53 for degradation (Sheng et al., 2006). Interestingly, tight control of p53 levels in muscle progenitors is pivotal for the regulation of differentiation and return to quiescence (Flamini et al., 2018), leaving open the question regarding USP7’s role during this process. USP7 can also regulate gene expression through its interactions with Polycomb Repressor Complex (PRC) components and other epigenetic regulators (Inoue et al., 2015; Lecona et al., 2015; Yamaguchi et al., 2017; Maat et al., 2021). Thus, it is possible to speculate that USP7 promotes differentiation by 1) controlling p53 levels, 2) interacting with specific transcriptional regulators such as Myogenin, and 3) by stablishing a favorable epigenetic landscape for the muscle genetic program.

These are just two examples highlighting the crosstalk between the UPS and signaling pathways controlling fate decisions in SCs. Future studies focusing on the UPS regulation, including proteasome assembly/composition and control of E3s/DUBs activity and expression, could allow a deeper understanding of SC heterogeneity, how this impact the process of muscle regeneration and the long-term maintenance of a functional SC pool.

In the context of pathological loss of muscle mass and aging, it becomes evident that UPS regulation can differentially impact myofibers and SCs; since approaches to manipulate the UPS move forward for the treatment of different diseases, more research is needed from the SC field to determine the effects over skeletal muscle maintenance and repair in the long run.
